# The Carrier Frequency of Two *SMN1* Genes in Parents of Symptomatic Children with SMA and the Significance of *SMN1* Exon 8 in Carriers

**DOI:** 10.3390/genes14071403

**Published:** 2023-07-06

**Authors:** Joanne E Davidson, Jacqueline S Russell, Noelia Nunez Martinez, David R Mowat, Kristi J Jones, Edwin P Kirk, Didu Kariyawasam, Michelle Farrar, Arlene D’Silva

**Affiliations:** 1 Department of Neurology, Sydney Children’s Hospitals Network, Sydney, NSW 2031, Australia; joanne.davidson1@health.nsw.gov.au (J.E.D.);; 2 Centre for Clinical Genetics, Sydney Children’s Hospital, Randwick, Sydney, NSW 2031, Australia; jacqui.russell@health.nsw.gov.au (J.S.R.);; 3 Department of Clinical Genetics, The Children’s Hospital at Westmead, and Discipline of Paediatrics and Child Health, University of Sydney, Sydney, NSW 2006, Australia; 4 Discipline of Paediatrics and Child Health, School of Clinical Medicine, University of New South Wales Sydney, Sydney, NSW 2052, Australia

**Keywords:** spinal muscular atrophy, carrier frequency, silent carrier, reproductive carrier screening

## Abstract

Background: Current carrier screening methods do not identify a proportion of carriers that may have children affected by spinal muscular atrophy (SMA). Additional genetic data is essential to inform accurate risk assessment and genetic counselling of SMA carriers. This study aims to quantify the various genotypes among parents of children with SMA. Method: A retrospective cohort study was undertaken at Sydney Children’s Hospital Network, the major SMA referral centre for New South Wales, Australia. Participants included children with genetically confirmed SMA born between 2005 and 2021. Data was collected on parent genotype inclusive of copy number of *SMN1* exons 7 and 8. The number of *SMN2* exon 7 copies were recorded for the affected children. Descriptive statistics were used to determine the proportion of carriers of 2+0 genotype classified as silent carriers. Chi-square test was used to correlate the association between parents with a heterozygous *SMN1* exon 7 deletion and two copies of exon 8 and ≥3 *SMN2* copy number in the proband. Results: SMA carrier testing was performed in 118/154 (76.6%) parents, incorporating 59 probands with homozygous *SMN1* deletions and one proband with compound heterozygote pathogenic variants. Among parents with a child with SMA, 7.6% had two copies of *SMN1* exon 7. When only probands with a homozygous *SMN1* exon 7 deletion were included, 6.9% of parents had two copies of *SMN1* exon 7. An association was observed between heterozygous deletion of *SMN1* exon 7 with two copies of exon 8 in a parent and ≥3 *SMN2* copy number in the affected proband (*p* = 0.07). Conclusions: This study confirmed a small but substantial proportion of silent carriers not identified by conventional screening within an Australian context. Accordingly, the effectiveness of carrier screening for SMA is linked with genetic counselling to enable health literacy regarding high and low risk results and is complemented by new-born screening and maintaining clinical awareness for SMA. Gene conversion events may underpin the associations between parent carrier status and proband *SMN2* copy number.

## 1. Introduction

Spinal muscular atrophy (SMA) is an autosomal recessive neuromuscular disorder characterised by progressive muscle weakness. Without therapeutic intervention, infantile onset (type 1) SMA is associated with a 95% mortality rate at two years of age [1]. However, targeted genetic therapies have revolutionised SMA care, improving the survival and reducing the comorbidities associated with the disease. With the introduction of reproductive genetic carrier screening (RCS) for SMA there is an opportunity for couples to become aware of their reproductive risk of having an affected child. Beyond the restoration of reproductive confidence, it remains vital that prospective parents have complete information including the burden, cost, and barriers to access treatment, to fully inform their reproductive decision-making [2].

SMA is caused by biallelic disruption of the survival motor neuron 1 (*SMN1)* gene, leading to inadequate levels of survival motor neuron (SMN) protein, which is essential to maintain the integrity and survival of anterior motor neurons [3,4]. Approximately 95% of SMA cases are due to homozygous deletion of exon 7 in the *SMN1* gene. Compound heterozygosity of an *SMN1* exon 7 deletion in trans with a pathogenic *SMN1* sequence variant accounts for a further 5% of cases [3,5]. A paralogous gene, survival motor neuron 2 (*SMN2*), which differs from *SMN1* by five base pairs within the coding region, modulates the phenotype in a dose-dependent manner and is the most significant predictive biomarker of disease severity [6].

Reproductive genetic carrier screening (RCS) for SMA is generally limited to assays including multiplex ligation-dependent probe amplification (MLPA) or quantitative polymerase chain reaction (qPCR) that determine the copy number of *SMN1* exon 7 (referred to as 1+0 genotype) [7]. These conventional and currently utilised methods do not detect sequence variants and will not identify deletion carriers who have two copies of *SMN1* on the non-deleted chromosome (referred to as 2+0 genotype) and those with a pathogenic *SMN1* sequence variant (1+1^D^ or 2+1^D^ genotype) [7]. Individuals with sequence variants or a 2+0 genotype are referred to as ‘silent carriers’, appearing as an *SMN1* copy number of 2 on MLPA testing, resulting in the potential to falsely reassure parents of a non-carrier status [8,9]. Rare cases of gonadal and somatic mosaicism have also been reported [10,11]. The presence of a 1+0, 2+0, or 1+1^D^ genotype significantly impacts the chance of recurrence for a couple with a previous affected infant who has homozygous deleted *SMN1* genotype.

Within the differing incidence of SMA internationally, the epidemiology of silent carriers also varies across jurisdictions with an increased incidence noted in certain African and Asian subgroups [12,13]. Within an Australian context, the epidemiology of silent carriers of SMA has been historically ascertained [14]. However, within an evolving diagnostic landscape where families have access to RCS and newborn screening, there is an imperative to investigate the contemporary epidemiology of silent carriers to inform local population screening.

In addition to epidemiological considerations, genotype as informed by carrier screening has the potential to affect SMA phenotype severity between generations. Gene conversion events, occurring at the c.840C>T nucleotide in *SMN*, are postulated to be relatively common, driving an increase in *SMN2* copy number. In SMA-affected individuals, with bi-allelic *SMN1* exon 7 deletion, the presence of *SMN1* exon 8 is associated with both a greater *SMN2* exon 7 copy number [15,16,17,18,19,20,21], as well as the translocation of an *SMN2* exon 7 copy to the telomeric *SMN1* region; both suggestive that the presence of *SMN1* exon 8 reflects a gene conversion event of *SMN1* exon 7 to *SMN2* exon 7 rather than a simple deletion. Consequently, a carrier with a heterozygous deletion of *SMN1* exon 7 and two copies of exon 8 may have a child with an increase in *SMN2* copy number, thus predicting a less severely affected child.

## 2. Aims

1. To investigate the genotype of SMA carriers using current carrier screening techniques. We aim to determine the proportion of silent carriers (2+0 and 1+1^D^) in an Australian state-based population and compare this to international data;
2. To assess the relationship between parental *SMN1* exon 8 copy number and proband genotype.

## 3. Methods

### 3.1. Study Design and Participants

This was a retrospective cohort study including carrier parents of children with SMN-related SMA (0 to 18 years) born between January 2005 and December 2021, referred, and managed at the Sydney Children’s Hospital Network state-wide tertiary neuromuscular clinic. This time-period was chosen to reflect when routine carrier genetic testing commenced with the introduction of MLPA technology. Genetic testing was undertaken within the context of contemporary clinical practice and reported the number of copies of *SMN1* exons 7 and 8. Probands were excluded when one or neither parent had undertaken SMA carrier testing. In the case of an affected sibship, the elder sibling was considered as the proband, and parental carriers only included once within the study population. The study was approved by the Sydney Children’s Hospital Network Human Research Ethics Committee (2020/ETH02020).

Data was obtained from medical records and the state-wide clinical genetics database (Trakgene, software, version 2.7.19). Collated data included genotype, ethnicity, and family history of neuromuscular disease of the family unit (carriers and probands).

Genotype was determined by copy number of *SMN1* exons 7 and 8 in the proband and carriers, and *SMN2* exon 7 in the proband. DNA from parents and probands were tested for *SMN1* exon 7 and exon 8 copy number using the P060-B2 SMA MLPA kit produced by MRC-Holland. The *SMN1* exon 7 probe has its ligation site at the C-to-T transition in exon 7 (c.840C>T) while the SMN1 exon 8 probe is able to distinguish between *SMN1* and *SMN2* at exon 8 (G-to-A transition (c.1155G>A). The *SMN2* copy number quantitative analysis was performed by quantitative real time PCR and by droplet digital PCR from 2021 onwards.

**Figure 1:**
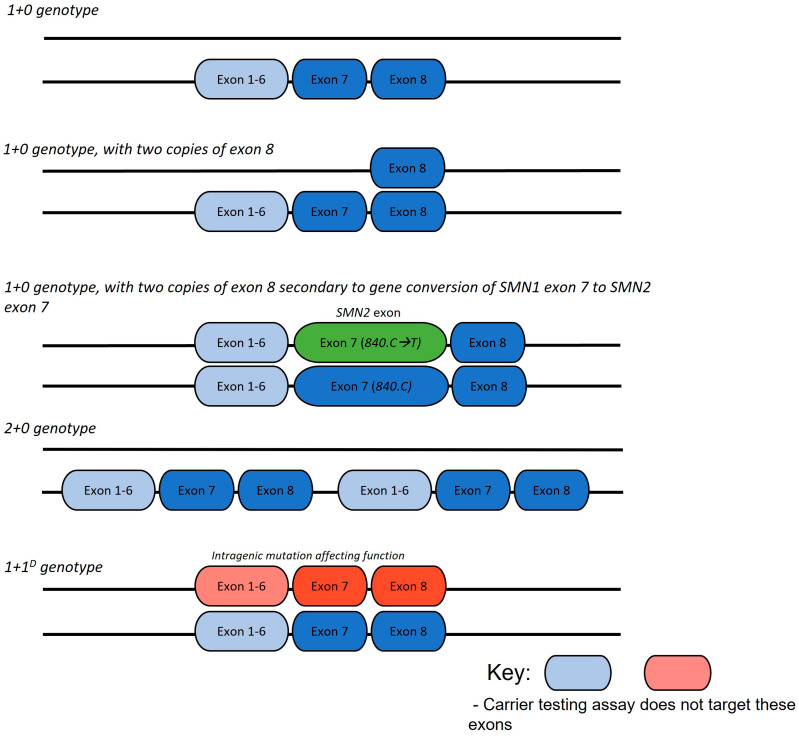
Common SMA carrier genotype arrangements.

The results of *SMN1* sequencing, if undertaken, were recorded for children with SMA. Any further genetic analysis that identified carrier status was collected, including linkage analysis, functional RNA studies, genetic data from siblings or identification of intragenic mutations through research methods (including DNA sequencing). Specific parental carrier status and genotype were ascribed from analysis (Figure 1).

### 3.2. Literature Review

To allow for comparison with worldwide data, a literature review utilising two major medical databases, MEDLINE and PubMed, was conducted to identify any previously published studies that matched our methodology. We searched all articles available in English from January 1999 to October 2022 matching the search terms (SMA or spinal muscular atrophy) AND (Prenatal Diagnosis/or carrier screening.mp or Genetic Carrier Screening). We only included papers that incorporated the genetic data (*SMN1* copy number) of parents of affected children with SMA, excluding all studies that statistically analysed carrier frequencies of *SMN1* copy numbers within the general population to infer the percentage of silent carriers. All studies identified as eligible during abstract screening were then screened at a full-text stage by two reviewers (A.D. and J.D.). The full-text studies identified at this stage were included for the data extraction. Following reconciliation between the two investigators, a third reviewer (D.K.) was added to reach consensus for any remaining discrepancies.

Only studies that reported the number of parents with two *SMN1* copy numbers were included and percentages of *SMN1* exon 7 gene dosages were compiled. This method was chosen to allow for direct comparisons with previously published literature. Studies were segregated into those that included compound heterozygote probands and those with homozygous deletion of *SMN1*. To ensure a more accurate SMA carrier rate for risk analysis, we combined our population data with the published data.

### 3.3. Statistical Analysis

Clinical and genetic data were analysed using descriptive statistics, including frequencies and percentages of *SMN1* exon 7 gene dosage, genotype, and carrier status for parents. Standard deviation and 95% confidence interval of previously published studies was calculated using Graph Pad Prism to compare our data to worldwide published data. The results for those that included compound heterozygote probands were adjusted, with the parents of these probands removed to allow for direct comparison. Categorical and non-parametric data were analysed using a pairwise Chi-square test to examine the association between a carrier with a heterozygous deletion of *SMN1* exon 7 with two copies of exon 8 and an *SMN2* exon 7 copy number of ≥3 in the proband. A *p*-value of 0.05 was used to determine the statistical significance. Statistical analyses were performed using the GraphPad QuickCalcs Web site: http://www.graphpad.com/quickcalcs/ConfInterval1.cfm (accessed on 20 December 2022).

## 4. Results

**Figure 2:**
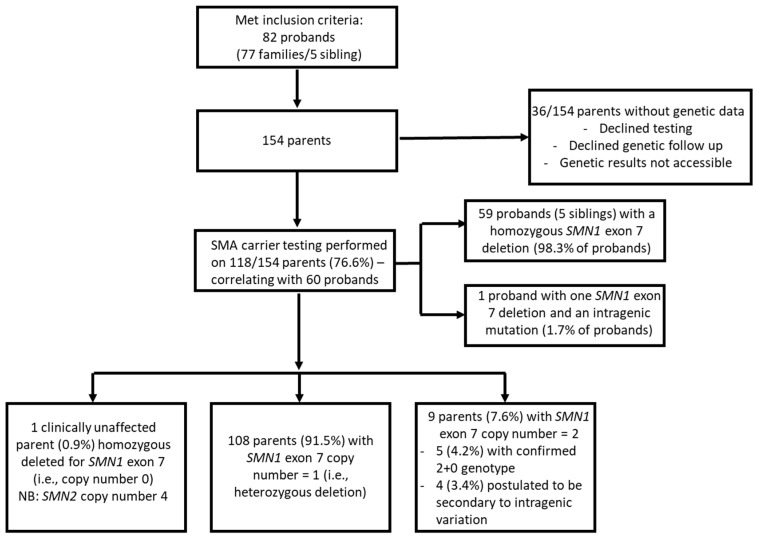
Flowchart of spinal muscular atrophy carrier screening testing in New South Wales Australian population.

The cohort consisted of 82 probands from 77 families. SMA carrier testing was performed in 118/154 (76.6%) parents of 60 probands. Genetic data was unavailable for 36 parents due to declining testing for personal reasons, testing performed in external laboratories where results were not accessible, and parents lost to follow-up (Figure 2).

Of the 118 parents, 116 (98.3%) were parents of 59 probands with homozygous deletion of *SMN1* (0+0), while two (1.7%) were parents of 1 proband who had a compound heterozygous genotype involving *SMN1* exon 7 deletion and an intragenic point mutation (0+1^D^).

Parental carrier screening identified two copies of *SMN1* exon 7 in 9/118 (7.6%) parents and within this population. This subgroup includes two probands diagnosed through NBS.

**Figure 3:**
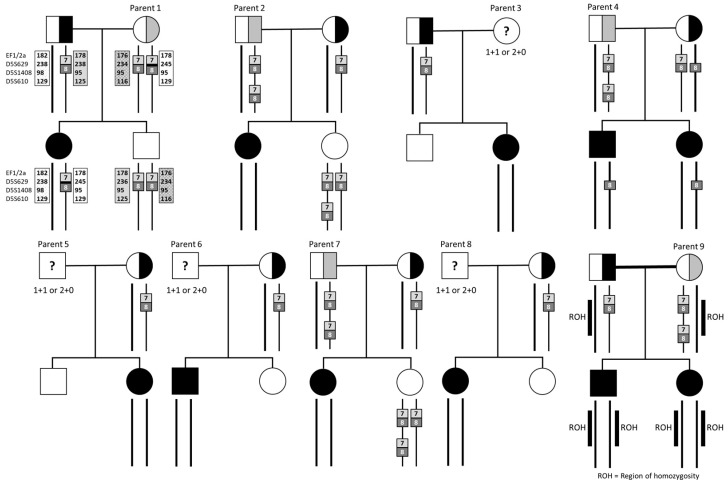
Pedigrees, gene dosages, and analysis of nine families. All nine parents of the undetermined genotype had children with homozygous *SMN1* deletion. Square: males, circles: females.

Out of the population, there was one copy of *SMN1* exon 7 in 108/118 (91.5%) parents, and one parent who was clinically unaffected despite homozygous deletion of *SMN1* and was later confirmed to have four copies of *SMN2*, likely serving to moderate clinical phenotype (Figure 3).

### 4.1. Parents with Two Copies of SMN1 Exon 7

Of the nine parents with two copies of *SMN1* exon 7, three (33.3%) were mothers and six (67.7%) were fathers. Of all parents with two copies of the *SMN1* gene, 7/9 (77.8%) were of European ancestry, 1/9 (11.1%) was of East Asian (Chinese), and 1/9 (11.1%) was of South Asian (Indian) heritage (Table 1, Figure 2). We were able to further clarify the genotype of five of the nine parents and determine that they were carriers following analysis of other family members. Parent 1 was inferred to have a pathogenic variant (1+1^D^) (1/118, 0.9%), and parents 2, 4, 7, and 9 (three fathers and one mother) were inferred to have the 2+0 genotype (4/118, 3.4%) after having further affected children, or through additional segregation in the family. Cascade testing was not undertaken in the other four parents and their genotype was undetermined (three fathers and one mother), and it remains possible that their child’s SMA may be due to a de novo deletion or non-paternity, in the case of the three fathers.

Specific cases highlighted the heterogeneity of carrier genotype in SMA, and the complex pathways required to elucidate carrier status. Parent 1 had two copies of *SMN1* exons 7 and 8, and Sanger sequencing did not identify point mutations in either allele. Transcriptomic studies of the trio showed no expression of *SMN1* mRNA in the proband and reduction in both parents, inferring a pathogenic variant, not identified with these approaches (Figure 2). Linkage analysis [15] showed that the affected proband had inherited one maternal haplotype (the presumed mutated copy) while an unaffected sibling had inherited the other haplotype, linked to a functional copy. Parents 2 and 7 were both identified as having 2+0 genotype based on the genetic analysis of another unaffected child who had inherited three *SMN1* gene copies. Parent 4 was identified as a likely 2+0 genotype carrier after having two affected children with no *SMN1* gene copies. Although gonadal mosaicism could not be ruled out, a de novo mutation is less likely. Parent 9 was a mother of two affected children. The parents were first cousins and the single nucleotide polymorphism (SNP) array of both children detected multiple regions of homozygosity consistent with parental consanguinity. A large 51 Mb region of homozygosity on chromosome 5 encompassing both the *SMN1* and *SMN2* genes indicated that the proband and the sibling had inherited a shared haplotype from both parents in this region, thus implying that Parent 9 had a 2+0 genotype (Figure 2).

### 4.2. Results in the Context of Worldwide Published Literature

The literature review identified two comparable studies [16,17] (Table 2) including probands of all genotypes (homozygous deletions and compound heterozygotes) and three comparable studies [14,18,19] including only probands with a homozygous *SMN1* exon 7 deletion (Table 3). In all studies including probands with an *SMN1* exon 7 homozygous deletion and compound heterozygotes, 16/227 parents (7.1%) were observed to have two *SMN1* exon 7 copies.

In comparison to the cumulative findings of prior international studies, where only probands with homozygous deletions were included, the current study had a higher rate of individuals with two copies of *SMN1* exon 7 (the current study had 8/116 (6.9%) versus the cumulative of prior studies 36/808 (4.5%), χ2 = 1.33, *p* = 0.24). However, this rate of 6.9% was comparable to the previous Australasian study rate of 6.0% [14] (Table 2).

### 4.3. Analysis of Parents with a Heterozygous Deletion of SMN1 Exon 7 and Two Copies of Exon 8

This study identified 52 probands born in 2009 or later with *SMN2* data available. A total of 42 probands and 84 parents were included in the further analysis. Exclusion of 10 probands occurred secondary to missing genetic information in 1 parent, and absence of exon 8 information occurred in the remainder.

The parents of 6/42 (14.2%) probands had a heterozygous deletion of *SMN1* exon 7 and two copies of exon 8. All six probands had ≥3 *SMN2* copies. The unidirectional association of at least one parent who was heterozygous for *SMN1,* with two copies of exon 8 and an *SMN2* copy number of three or more in the proband was χ^2^ = 3.231 (*p* = 0.07) (Table 4).

## 5. Discussion

Spinal muscular atrophy, even within the new post treatment era continues to confer a substantial morbidity for affected individuals and remains a devastating diagnosis for families. This study presents the evaluation of the genotypes amongst parents of children with SMN-related SMA in a state-based Australian population within a paradigm of genetic carrier and newborn screening (NBS) and disease modifying therapy for SMA. The results highlight that current screening methods (MLPA or qPCR) do not identify a small but significant proportion of the SMA carrier population who may have children with SMA. This may be due to parents having a 2+0 genotype or a *SMN1* point mutation, or when SMA arises from a de novo variant (exon 7 deletion). These findings have important implications for healthcare, emphasising the need to support health literacy in order to understand the low and high-risk results within screening programmes. Furthermore, NBS compliments carrier screening, to identify affected children early and together provide primary and secondary preventative strategies [22,23]. However, current NBS testing strategies used in Australia will not identify point mutations, or 2+0 genotype carriers which means that a small gap remains. In addition, this study observes a possible association between carrier parents (with a heterozygous deletion of *SMN1* exon 7 and two copies of exon 8) and probands with ≥3 *SMN2* copy numbers with the potential to confer a milder phenotype, postulated to be secondary to gene conversion events. The rate of silent SMA carriers can also be studied through assessment of population allele frequencies of 1, 2, and 3 copies of *SMN1,* inferring the percentage of 2+0 genotype utilising the principles of the Hardy–Weinberg equilibrium. In a 2014 meta-analysis using pooled data of 169,000 individuals in 14 published studies, this method estimated the 2+0 genotype to occur in 3.1% of Caucasian-heritage populations, 4.1% of Asian-heritage populations (defined as), 27.9% of African-heritage populations, 7.4% of Ashkenazi Jewish populations, 7.8% of Hispanic-heritage populations, and 8.1% of Asian Indian-heritage populations [14,20,24]. While these are comparable to the observed frequencies of 2+0 genotype ascertained by the methodology in the current study, the variations in the reported rates recapitulate the challenges shared by many rare diseases. Among these are small patient numbers and lack of a coordinated strategy for data mining. Whilst methodologies that ascertain population allele frequencies use large databases, thus counteracting the potential biases associated with the small sample size, they provide limited insight into the frequency of intragenic mutations contributing to a silent carrier status, relying on an assumed frequency of this genotype [24,25].

Ethnicity is noted to be associated with variation in silent carrier rate, however with a focus on the predominant ethnic groups within a population. Of our silent carrier cohort, 22% were of non-European background and identified as 2+0 genotype. Our study is limited in analysing silent carrier status within ethnic subgroups, which may limit the generalisability to broader populations. For example, the role of Australia’s unique ethnic spectrum may warrant further studies on the prevalence of the 2+0 genotype in broader subpopulations, including Indigenous subgroups to support equity of reproductive choice [13,21].

Within Australia, the rate of silent carriers has remained unchanged over the last two decades [14], yet continues to be higher when compared to similarly conducted international studies that have focussed on determining parental silent carrier status retrospectively from the birth of an affected child. As can be seen in our study, the recurrence risk for subsequent pregnancies is high in parents with the ‘2+0’ genotype with the potential to lead to several affected children within a sibship. In contrast, in the four parents with two copies of *SMN1* exon 7 without further clarification (parents 3, 5, 6, and 8), it remains possible that the SMA in their child occurred as a de novo event, where the parent is truly not a carrier, with a low chance of recurrence in future pregnancies. If the previously published de novo rate of 2% [26] holds true for our cohort, it is plausible that at least one of these parents are not carriers. Thus, it remains imperative to distinguish between these two possibilities (of a de novo vs. a 2+0 genotype) through extended analysis of SMN, as it directly informs genetic counselling and decisions surrounding risk in future pregnancies.

As noted within our silent carrier cohort who had a spectrum of methodologies used to accurately determine carrier genotype, a multi-dimensional methodological approach to ascertaining carrier haplotype may be necessary. This includes utilising approaches including but not limited to SMN gene dosage, extending to linkage analysis, whole genome sequencing, long read sequencing, and RNA expression techniques. Thus, integration and collaboration between clinical and genetic laboratory testing services, to understand the clinical scenario including details of a comprehensive pedigree, coupled with expertise in diagnostic genomics will help navigate the complexities of SMN carrier elucidation, especially for individuals with a silent carrier status.

Through our findings, an association was observed between carrier parents with a heterozygous deletion of *SMN1* exon 7 and two copies of exon 8 and a prognosticated milder genotype in the SMA-affected child (denoted by three or more ≥*SMN2* exon 7 copy numbers). This observation supports the concept that the presence of *SMN1* exon 8 in the absence of exon 7 may indicate an *SMN2* exon 7 gene conversion event. Further study of the manner of architectural rearrangement and the rate of gene conversion events between generations may have clinical relevance to facilitate stratification of the severity of phenotype for subsequent pregnancies.

Whilst this study provides a retrospective snapshot, with technological advances being implemented in health practice and policy, the SMA clinical paradigm continues to rapidly change. In this context, the Australian Federal government has announced that reproductive carrier screening for SMA will be placed on the Medicare Benefit Schedule in November 2023, providing options for couples on their reproductive journey and reducing inequities of having to pay for screening. Accordingly, the present study provides critical data for its implementation, confirming that reproductive screening cannot stand alone and requires a model of care inclusive of robust bi-directional links across screening, diagnostic, and clinical (including genetic counselling) services, to ensure a patient- and family-focused delivery of screening processes [27]. Collectively, disease modifying therapies, NBS and RCS enable a precision medicine model of care. The awareness among health practitioners regarding predictive factors of SMA severity, such as *SMN2* copy number and polymorphisms, as well as knowledge about therapeutic and reproductive options are important to provide optimal support and guidance to parents who receive high-risk screening results and can assist them in making informed decisions.

This study has some limitations in its applicability to a general and Australian population. The retrospective dataset was susceptible to ascertainment bias as prior to 2009 carrier testing of parents of SMA-affected children was not routinely offered. The data was also limited by the number of parents with 2+0 genotypes who proceeded with or were offered further testing based on clinical need and contemporary technologies to determine the genetic aetiology of their silent carrier status. The prospect of incorporating next generation sequencing (NGS) into carrier screening workflows to accurately identify silent carriers has been postulated [28]. In support of this notion, a recent economic evaluation of population-based expanded reproductive carrier screening for 300 recessive genes using a NGS panel demonstrated the cost effectiveness from health service and societal perspectives [29]. However, the high homology between *SMN1* and *SMN2* leads to poor mappability for short read sequencing methods (Table 5). Current available testing methodologies would not allow cost-effective identification of point-mutations in the screening context. However, as costs fall, this may change.

## Figures and Tables

**Table 1:** Parental genotypes corresponding to ethnic heritage.

Genetic Heritage	Total Number of Parents with Genetic Data	Parents with Two *SMN1* Copies	2+0 Genotype	Point Mutation	Not Determined
Caucasian	76 (64.4%)	7/76 (9.2%)	2/76 (2.6%)	1/76 (1.3%)	4/76 (5.3%)
Hispanic	1 (0.8%)	0	-	-	-
East Asian ^1^	6 (5.1%)	1/6 (16.7%)	1/6 (16.7%)	-	-
South Asian ^2^	14 (11.9%)	1/14 (7.1%)	1/14 (7.1%)	-	-
West Asian ^3^	14 (11.9%)	0	-	-	-
African	5 (4.2%)	0	-	-	-
Polynesian	2 (1.7%)	0	-	-	-

Frequencies of parental genotype for different ethnicities are presented as n (%) with different denominators used to illustrate the percentage of silent carriers within each ethnic subtype. ^1^ Defined as those with ethnic origins in the modern states of China, Japan, Mongolia, North Korea, South Korea, or Taiwan. ^2^ Defined as those with ethnic origins in the modern states of Afghanistan, Bangladesh, Bhutan, India, Maldives, Pakistan, and Sri Lanka. ^3^ Defined as those with ethnic origins in the modern regions of the Arabian Peninsula, Caucasus Region, Iran, Mesopotamia, Fertile Crescent, Cyprus, Turkey, and Egypt.

**Table 2:** A summary of previous English-language studies and this study included probands of all genotypes.

Author	Population	Proband Genetics	Number Analysed	Result	Genotype
Sheng-Yuan et al. (2010) [16]	Chinese	8% compound heterozygote 92% homozygous deletion	44 parents	4/44 (9.1%) had two *SMN1* exon 7 copies	2 (4.5%) intragenic mutation 2 (4.5%) 2+0 genotype
Ar Rochmah et al. (2017) [17]	Japanese	3% compound heterozygote 97% homozygous deletion	65 parents	3/65 (4.6%) had two *SMN1* exon 7 copies	1 (1.5%) intragenic mutation 1 (1.5%) 2+0 genotype 1 (1.5%) not further studied
**Total Combined**		6.4% (7/109) had 2 *SMN1* exon 7 copies			
This Study	Australian (NSW)	1.7% compound heterozygote 98.3% homozygous deletion	118 parents	9/118 (7.6%) had 2 *SMN1* exon 7 copies	1 (0.9%) intragenic mutation 4 (3.4%) probable 2+0 genotype 4 (3.4%) not further studied
**Total Combined**		16/227 (7.1%) had 2 *SMN1* exon 7 copies			

**Table 3:** A summary of previous English-language studies and this study including only probands with a homozygous *SMN1* exon 7 deletion.

Author	Population	Number Analysed	Result	Genotype
Mailman et al. (2001) [18]	North American	100 parents	4/100 (4.0%) had 2 *SMN1* exon 7 copies	1 (1%) 2+0 genotype 1 (1%) de novo mutation 2 (2%) not further studied
Smith et al. (2007) [14]	Australian (Victoria)	117 parents	7/117 (6.0%) had 2 *SMN1* exon 7 copies	2 (1.7%) 2+0 genotype 2 (1.7%) de novo mutation 3 (2.6%) undetermined
Sheng-Yuan et al. (2010) [16]	Chinese	40 parents	2/40 (5.0%) had 2 *SMN1* exon 7 copies	2 (5.0%) 2+0 genotype
Alias et al. (2014) [19]	Spanish	488 parents	21/488 (4.3%) had 2 *SMN1* exon 7 copies	15 (3.1%) 2+0 genotype 5 (1.0%) de novo mutation 1 (0.2%) undetermined
Ar Rochmah et al. (2017) [17]	Japanese	63 parents	2/63 (3.2%) had 2 *SMN1* exon 7 copies	1 (1.6%) 2+0 genotype 1 (1.6%) not further studied
**Statistics**		Median: 4.5%, Mean: 4.5%, SD: 1.05%, 95% CI: 3.63–5.46%		
**Total Combined**		36/808 (4.5%) had 2 *SMN1* exon 7 copies		
This study	Australian (NSW)	116 parents	8/116 (6.9%) had 2 *SMN1* exon 7 copies	3 (2.6%) probable 2+0 genotype 5 (4.3%) not further studied
**Total Combined**		44/924 (4.8%) had 2 *SMN1* exon 7 copies		

**Table 4:** Analysis of *SMN2* copy number and parental genotype of heterozygous deletion of *SMN1* exon 7 and two copies of exon 8.

	Probands with Two or Less *SMN2* Copies	Probands with Three or More *SMN2* Copies	Total
**Carrier with a 1:1 copy number ratio of *SMN1* exon 7 to exon 8**	15	21	36
**Carrier with heterozygous deletion of *SMN1* exon 7 and 2 copies exon 8**	0	6	6
**Total**	15	27	42

χ^2^ statistic = 3.231. *p* = 0.07.

**Table 5:** A summary of studies aimed to detect silent carriers.

Author	Methods	Result
Luo et al. (2014) [30]	Microsatellite analysis identified haplotype blocks and next-generation sequencing identified specific SNPs associated with the 2+0 genotype in those with Ashkenazi Jewish heritage.	Identified g.27134T>G in intron 7 and g.27706_27707delAT in exon 8 as polymorphisms associated with 2+0 carrier status. This also had predictive value in those of an African American, Asian, Hispanic, and Caucasian background.
Alias et al. (2018) [31]	Examined g.27134T>G and g.27706_27707delAT in a Spanish population	It was found that the presence of the SNPs increased or reduced the residual carrier risk in this population.
**PCR-Based Technologies**		
Vidal-Folch et al. (2018) [32]	Used digital droplet PCR to simultaneously screen for *SMN1* and *SMN2* copy numbers as well as the g.27134T>G SNP.	Allowed for accurate screening of carriers without requiring standard curves. Interassay imprecision was <7.1% CV and interassay imprecision was <6.0% CV. Testing was 100% specific and sensitive in SMA.
Azad et al. (2020) [33]	Custom SNP-specific assay using TaqMan genotyping technology to determine CNV of *SMN1, SMN2* and presence of g.27134T>G SNP	Results are 100% concordant with standard PCR in 21 pilot samples. Good reproducibility with a 1–4% CV for all genotypes.
**Next-Generation Sequencing Technologies**		
Feng et al. (2017) [34]	Paralogous gene copy-number analysis by ratio and sum to detect *SMN1, SMN2* copy numbers, and g.27134T>G SNP on short-read next-generation sequence	Results are 100% sensitive and 99.6% specific to detect 1 copy of *SMN1*, and 100% concordant detection of g.27134T>G SNP compared to RFLP assay. Carrier detection rates increased according to ethnic heritage; African American (70.5% to 90.3%), Ashkenazi Jewish (90.5% to 92.8%), Asian (93.3% to 93.6%), Caucasian (94.8% to 95%), and Hispanic (90% to 92.6%)
Ceylan et al. (2020) [35]	Used CODE-SEQ technology; an NGS assay of 18 pairs of coded oligonucleotides coupled with a unique probe to target SMA-related loci and reference regions	Results show 100% correlation with the MLPA results in all 80 samples tested for exon 7 *SMN1* CN. Unable to test the accuracy in the detection of the g.27134T>G SNP as none were present in the sample.
Chen et al. (2020) [36]	Analysed read depth and used eight reference genome differences to identify copy number of *SMN1* and *SMN2* as well as g.27134T>G SNP.	Accuracy of 99.8% and 99.7% for *SMN1* and *SMN2* CN compared to qPCR and MLPA, precision of 100% for both SMA and 1+0 carrier status. Carrier detection rates increased with SNP by 21.3% in those with African heritage and 2% at most for all other heritages.
Li et al. (2022) [28]	Utilised long-range PCR and third-generation sequencing of full-length and downstream regions of *SMN1* and *SMN2.*	Improved detection rates of SMA carriers in a Chinese population from 91% to 98%, including three *SMN1* intragenic mutations and an in-frame mutation to *SMN2*.

## Data Availability

The data presented in this study are available on request from the corresponding author. The data are not publicly available due to maintaining confidentiality.
